# Species Distribution Models of the *Spartina alterniflora* Loisel in Its Origin and Invasive Country Reveal an Ecological Niche Shift

**DOI:** 10.3389/fpls.2021.738769

**Published:** 2021-10-12

**Authors:** Yingdan Yuan, Xinggang Tang, Mingyue Liu, Xiaofei Liu, Jun Tao

**Affiliations:** ^1^Jiangsu Key Laboratory of Crop Genetics and Physiology, College of Horticulture and Plant Protection, Yangzhou University, Yangzhou, China; ^2^Co-innovation Center for Sustainable Forestry in Southern China, Jiangsu Province Key Laboratory of Soil and Water Conservation and Ecological Restoration, Nanjing Forestry University, Nanjing, China; ^3^College of Mining Engineering, North China University of Science and Technology, Tangshan, China; ^4^Institute of International Rivers and Eco-Security, Yunnan University, Kunming, China

**Keywords:** *Spartina alterniflora*, invasive country, origin country, MaxEnt model, niche shift

## Abstract

*Spartina alterniflora* is a perennial herb native to the American Atlantic coast and is the dominant plant in coastal intertidal wetlands. Since its introduction to China in 1979, it has quickly spread along the coast and has caused various hazards. To control the further spread of *S. alterniflora* in China, we first reconstructed the history of the spread of *S. alterniflora* in its invasion and origin countries. We found that *S. alterniflora* spreads from the central coast to both sides of the coast in China, while it spreads from the west coast to the east coast in America. Furthermore, by comparing 19 environmental variables of *S. alterniflora* in its invasion and origin countries, it was found that *S. alterniflora* is more and more adaptable to the high temperature and dry environment in the invasion country. Finally, we predicted the suitable areas for this species in China and America using the maximum entropy (MaxEnt) model and ArcGIS. Overall, through analysis on the dynamic and trend of environmental characteristics during the invasion of *S. alterniflora* and predicting its suitable area in the invasion area, it guides preventing its reintroduction and preventing its further spread of the species has been found. It has reference significance for studying other similar alien plants and essential enlightening relevance to its invasion and spread in similar areas.

## Introduction

Biological invasion is considered the second-largest factor leading to the reduction of biodiversity after habitat fragmentation (Vitousek et al., 1996; Wilcove et al., 1998). Plant invasion is an important branch of biological invasion research, which refers to the phenomenon where an alien plant is introduced into other new areas by human or natural factors; then, after some time, it can grow and reproduce wildly and threaten the local ecosystem, biodiversity, and even human health (Mack et al., 2000; Sakai et al., 2001; Richardson and Van Wilgen, 2004). Meanwhile, this plant is called an invasive plant. Invasive alien plants can quickly replace native species, change the structure and function of native communities, and affect ecosystem processes (Tilman, 2000; Raghubanshi and Tripathi, 2009). China is one of the critical victims of biological invasions (Raghubanshi and Tripathi, 2009; Zhu, 2012). China has more than 30 invasive species among the 100 most threatened invasive species around the world as announced by the International Union for Conservation of Nature (IUCN) in 2000 (Tilman, 2000; Luque et al., 2013). The plant invasion in China has the characteristics of rapid spread, a wide hazard area, and an increasing number of new invasive species, which has caused huge economic losses to Chinese agriculture, forestry, animal husbandry, and fishery (Ding et al., 2008). Statistics showed that China needs to invest a lot of money to control these invasive plants (Lin et al., 2007; Weber et al., 2008).

A series of global changes caused by human activities have brought extremely favorable conditions for introducing and invading invasive plants (Walther et al., 2009). Several studies have considered global change and biological invasion in a comprehensive manner. Among them, changes in atmospheric composition (such as increased concentrations of CO_2_, O_3_, etc.), climate change, and nitrogen deposition have pronounced and more direct effects on the invasion process of alien plants (Stachowicz et al., 2002; Bradley et al., 2009). Simultaneously, based on the different stages of biological invasion, global changes may affect biological invasion (Catford et al., 2012). For example, the increase in temperature has caused some alien species to successfully survive the winter in invading areas, and the mortality rate has been dramatically reduced (Walther et al., 2007; Kiritani, 2011). Furthermore, changes in the interrelationships between invading species, such as rising sea levels, increase the surrounding salinity, so invasive species with stronger salt-resistance and flooding resistance gradually become dominant in the inter-species competition process (Perry and Atkinson, 2009). In short, global change will not only promote pest invasion but also change its distribution pattern. The systematic investigation of the dynamics of the distribution of important invasive species in global climate change is of great significance for managing invasive species.

*Spartina alterniflora* Loisel. is a perennial herb native to the American Atlantic coast and is the dominant plant in coastal intertidal wetlands (Bertness, 1991; Feist and Simenstad, 2000; Ayres, 2004). It has a large root system, dense plant growth, and a good siltation-promoting effect. It can effectively slow down and control coastal erosion in its place of origin and provide rich food sources and habitats for fish, birds, and other animals (Simenstad and Thom, 1995). *S. alterniflora* has an excellent ability to promote the land formation, countries worldwide have introduced and planted it. It has become one of the most successful invasive plants in the estuarine salt marsh wetland ecosystem. Furthermore, *S. alterniflora* has strong adaptability and spreading ability (Daehler and Strong, 1994, 1996), and it is widely distributed in wetlands along the beach. At present, its distribution area has expanded from the Atlantic coast of North and South America to Europe, the west coast of North America, New Zealand, and the coast of China (Baumel et al., 2001), resulting in changes in the structure and function of the gulf ecosystem (Simenstad and Thom, 1995). In the areas invaded by *S. alterniflora*, local species are repelled, with strong inter-species competitiveness. It rapidly spreads to occupy the empty niche in the intertidal zone (Feist and Simenstad, 2000), resulting in the reduction of the beach area and the change of ecological environment, thereby reducing the distribution number and species of benthic organisms (Dumbauld et al., 1995), changing the original habitats of birds, and affecting the diversity of local birds (Evans, 1986). The proliferation of *S. alterniflora* in the invaded area has directly or indirectly reduced the biodiversity of the invaded area, which has brought significant losses to local biodiversity protection management and economic development (Chen et al., 2004; Chung et al., 2004; Li and Zhang, 2008). In particular, *S. alterniflora* was introduced into coastal wetlands of the China as an ecological project in 1979. It has strong adaptability in the coastal wetlands of the China, strong inter-species competition ability, rapid diffusion ability, and transmission power mechanism. Now, it is widely distributed in the coastal wetlands in China (Huang et al., 2008; Li and Zhang, 2008). Therefore, the large-scale spread of *S. alterniflora* in the coastal wetlands of China has caused losses to local environmental protection and economic development and has become one of the most invasive species in the coastal wetlands of China.

Biological invasion is essentially a dynamic process of species distribution (Dormann et al., 2012). The entire process of biological invasion is closely related to expanding the distribution area of alien species. The study of the dynamic diffusion of its distribution area has always been a critical issue in biological invasion. Therefore, more and more studies have been conducted on the historical patterns and potential ecologically suitable distributions of alien biological invasions (Salo, 2005; Wang and Wang, 2006). These studies further explore the possible routes of their spread from the reconstruction process of the history of alien species invasions. It is helpful to formulate effective prevention and control measures through the dynamic historical reconstruction of alien species distribution areas and the prediction of the spatiotemporal diffusion characteristics of species in potential distribution areas. As the niche shift of species changes with time and space, the phenomenon of a niche shift in alien species is particularly prominent (Müller-Schärer et al., 2004; Eastwood et al., 2007). Therefore, before predicting the potential ecologically suitable distributions of alien species, it is first necessary to determine whether the niche of the alien species in the invaded area has changed (Mitchell and Power, 2003; Dietz and Edwards, 2006). The current methods for the quantitative assessment of a niche overlap are increasingly becoming widely used in studying species niche changes over time and space, such as in *Nylanderia fulva* and *Pueraria montana* (Callen and Miller, 2015; Kumar et al., 2015). Therefore, studying the niche of the *S. alterniflora* origin and invasion countries is helpful to grasp the factors and thresholds that affect its distribution, as it is important to predict the potential distribution of *S. alterniflora*.

The species distribution model (SDM) is a modeling method that predicts the potential distribution of species based on the niche theory (Gelviz-Gelvez et al., 2015; Moor et al., 2015). There are many statements about the definition of a niche, the most famous of which is the multi-dimensional hypervolume niche model proposed by Hutchinson in 1957. In this study, it has been found that organisms in the environment result from the combined action of multiple environmental resource factors. Species have a specific suitable range for each environmental element, and the adaptation range of all ecological factors constitutes the ecological niche of the species in the environment (Perutz, 1967). Among the many species distribution models (Bioclim, Climex, Domain, Garp, and MaxEnt), the maximum entropy (MaxEnt) model is a density estimation and distribution prediction model based on the maximum entropy theory (Elith et al., 2006; Phillips et al., 2009). Its simulation accuracy is higher than other models, its prediction effect is better, and it is widely used (Phillips et al., 2009). The MaxEnt model calculates a prediction model based on the current distribution points of the species and the current climate data and then uses this model to simulate the distribution of the species in future climate states. Niche conservation is a theoretical premise that makes the simulation of species distribution in future climate valuable (Petitpierre et al., 2012). The ecologically suitable distributions for the most invasive species have been predicted using the MaxEnt model (O'donnell et al., 2012; Kelly et al., 2014).

At present, the research carried out on *S. alterniflora* has mainly focused on biology, ecological characteristics, competitive substitution mechanisms, and chemical control. However, it is not clear how *S. alterniflora* is introduced into China and its spread route and speed. In this study, we used MaxEnt modeling and bioclimatic data to characterize the niche shift of *S. alterniflora* between native and non-native countries. Our objectives were to: (1) understand the diffusion regularity of *S. alterniflora* in China and America and the stage of invasion in China; (2) compare the niche of *S. alterniflora* in the invasion and origin countries; (3) map the potential suitable distribution of *S. alterniflora* in China and America.

## Materials and Methods

### Species Occurrence and Reconstruction Historical Expansion Process of *S. alterniflora*

The distribution data for *S. alterniflora* in China were derived from the Global Biodiversity Information Facility (GBIF) database (http://www.gbif.org) and China Digital Herbarium (CVH, http://www.cvh.ac.cn/), supplemented with recent field surveys and literature records from China National Knowledge Infrastructure (http://www.cnki.net/) and Google Scholar. The distribution data for *S. alterniflora* in America were derived from the GBIF database (http://www.gbif.org) and literature records from Google Scholar. Duplicate latitude and longitude specimen information with unknown geographic coordinates were removed, and then the plant catalog of the sample plots was checked. For the available records that lacked latitude and longitude information, we used Google Earth to query the latitude and longitude information corresponding to the sample plots according to the place names. In this study, the limited number of distribution points were retained within a certain distance according to the spatial screening method, and systematic sampling was performed again according to the principle of uniformity and randomness to ensure that the geographic autocorrelation of the data points is minimized (Tang et al., 2021a). The distribution information of *S. alterniflora* in China (113 points) and in America (287 points) can be obtained and saved as a.csv file for later use. The climate variable data for this study were downloaded from the WorldClim database (http://www.worldclim.org/) from 1950 to 2000. We used the 19 bioclimatic variables at 30 arc seconds (about 1 km^2^) (Hijmans et al., 2005). The bioclimatic variables are shown in Table 1.

**Table 1:** Bioclimatic variables in this study.

**Type**	**Variables**	**Description**	**Units**
Bioclimatic variables	bio1	Annual mean temperature (°C)	°C
	bio2	Mean diurnal range [Mean of monthly (max temp–min temp)] (°C)	°C
	bio3	Isothermality (Bio2/Bio7) (×100)	–
	bio4	Temperature seasonality (standard deviation×100) (Coefficient of variation)	°C
	bio5	Max temperature of warmest month (°C)	°C
	bio6	Min temperature of coldest month (°C)	°C
	bio7	Temperature annual range (Bio5–Bio6) (°C)	°C
	bio8	Mean temperature of wettest quarter (°C)	°C
	bio9	Mean temperature of driest quarter (°C)	°C
	bio10	Mean temperature of warmest quarter (°C)	°C
	bio11	Mean temperature of coldest quarter (°C)	°C
	bio12	Annual precipitation (mm)	mm
	bio13	Precipitation of wettest month (mm)	mm
	bio14	Precipitation of driest month (mm)	mm
	bio15	Precipitation seasonality (coefficient of variation)	–
	bio16	Precipitation of wettest quarter (mm)	mm
	bio17	Precipitation of driest quarter (mm)	mm
	bio18	Precipitation of warmest quarter (mm)	mm
	bio19	Precipitation of coldest quarter (mm)	mm

The administrative division maps of China and America were downloaded from the National Science and Technology Infrastructure Platform (http://www.geodata.cn/). We used the geographic information system software ArcGIS to map the space-time diffusion process of *S. alterniflora* in the invading area of China and the country of origin, America.

### Selection and Comparison of Climate Variables

The results of the niche analysis and the prediction of the final ecologically suitable distributions directly impacted the selection of environmental variables. Therefore, the selection of appropriate environmental variables was a key link in the analysis of climate niches and the prediction of ecologically suitable distribution areas (Holm, 1979). To remove the high correlation between variables and reduce the over-fitting of the model, 19 environmental variables were extracted from all distribution points, and the correlation functions (ecospat.cor.plot, vifstep, princomp), and performed a correlation analysis (Pearson correlation coefficient, | *r* | ≥ 0.8). Meanwhile, we loaded 19 bioclimatic variables and species distribution points into the MaxEnt model and performed a modeling analysis based on default parameters. The contribution of the environmental variables was obtained from the MaxEnt model with default parameters. According to correlation analysis and the variable contribution rate, the variables with a small contribution in the high-correlation variable group should be removed (Li et al., 2020). In addition, the biological significance of the variables was fully considered. This process resulted in a set of environmental data containing seven environmental variables (bio2, bio3, bio5, bio8, bio12, bio14, and bio16) for America and six environmental variables (bio1, bio2, bio9, bio15, bio18, and bio19) for China. Note that, when studying the niche characteristics of an alien species during the invasion process, we usually choose to fix the native niche as a reference.

To roughly judge the similarities of the climatic conditions of China and the USA, we used SPSS 25 (IBM, Armonk, New York, USA) to compare the mean values of the 19 bioclimatic variables with a two-factor ANOVA without replication. We approximately distinguished the similarities and differences of the climatic conditions between the native and invasive countries. We used a bootstrapping procedure to determine whether differences in sample size affected univariate comparisons (Lee and Rodgers, 1998). We randomly sampled the same number of invasion distribution samples as the distribution points of origin and recalculated the mean and variance for each variable. This process was repeated 1,000 times, yielding a 95% CI for each environmental variable (*a* = 0.05) (Holm, 1979). The results of the comparison between the native and invasive countries were presented using a violin diagram.

### Bioclimatic Niche Shift

The distribution data in this study showed the current geographic distribution of *S. alterniflora* in China and America. Based on the geospatial distribution data of *S. alterniflora*, to make the selected background data reflect the actual distribution of the species, we used R and the ArcGIS 10.5 mapping software (Environmental Systems Research Institute, ESRI, Redlands, California, United States) R language 3.6.1 to apply the kernel density distribution map along with the diffusion rate of *S. alterniflora*. Around each distribution point, a buffer zone with a diameter of 1.5° was selected, and 10,000 points in each province buffer zone were chosen as its background data. Then, we extracted the climate data for each background point. The “ecospat” package was used to perform a principal component analysis (PCA)-env in R language.

We used the species niche climate spatial comparison method constructed by Broennimann to compare the niche characteristics of *S. alterniflora* in the countries of origin and invasion (Broennimann et al., 2012). The selected six available bioclimatic variables in America (bio2, bio3, bio5, bio8, bio12, bio14, and bio16) were used to perform the PCA-env method implemented by Broennimann. In addition, the quantified niche of a species in climate space was represented by some niche indices, such as the overlap index D, niche equivalence, and niche similarity. The niche overlap index D represented the overlap rate of species niche between two regions (Schoener, 1970), and its value ranged from 0 (the niche of the two regions does not overlap at all) to 1 (the niche of the two areas overlap entirely). The test of niche equivalence was conducted by collecting all distribution points, randomly reselecting distribution points equal to the initial number of origin and invasion sites in two regions, respectively, and calculating the D value. The random calculation was repeated 100 times, and the statistical test was performed for the niche overlap index *D* value (*a* = 0.05). The niche similarity test evaluated whether the degree of overlap between the observed niche in two regions is higher than the overlap between the experimental niche in one area and a randomly selected niche in the other region. The calculation was repeated 100 times randomly. Through the analysis of the niche stability, unfilled and expansion indicators in the entire background environment and the marginal climate of 75–100% background environment were found. This method automatically removed the sampling bias through a smooth kernel density function (Broennimann et al., 2012).

Each species was modeled to 10 times, and an average niche model was used to generate the predicted niche occupancy (PNO) profiles following Evans et al. (Evans et al., 2009). These profiles were estimated using single bioclimatic variables that summarize the annual conditions of the habitats of the species. We used the selected variables of America: mean diurnal range (Bio2), isothermality (Bio3), the maximum temperature of the warmest month (Bio5), mean temperature of the wettest quarter (Bio8), annual precipitation (Bio12), precipitation of driest month (Bio14), and precipitation of wettest quarter (Bio16). These analyses were performed with the R package ecospat.

### Predicting Ecologically Suitable Distributions of *S. alterniflora* in China and America

Based on the environmental variables of the actual distribution of *S. alterniflora* in China and America, the MaxEnt model was used to analyze the species niche demand and predict its potential distribution area (Yang et al., 2013). The specific operation method is as follows: First, the distribution occurrence data and selected environmental variables were loaded into the MaxEnt model. To estimate the capacity of the modeling, the running parameters were set to randomly select 25% of the distribution points as the test data and 75% of the distribution points as the training data for a model accuracy analysis. The maximum number of iterations is 500. Feature combination (FC) and regularization multiplier (RM) were optimized through calling the ENMeval packet in the R software, and other parameters were set as the default (Muscarella et al., 2014). There were many ways to divide ecological habitat suitability areas. The ecological habitat suitability areas of *S. alterniflora* were divided into four levels, namely, low habitat suitability (existence probability is <0.3), medium habitat suitability (existence probability is 0.3–0.5), high habitat suitability (existence probability is 0.5–0.7), and excellent habitat suitability (existence probability is >0.7).

The receiver operating characteristic (ROC) curve of the knife-cut method is an analytical method to evaluate the reliability of the model (Dorfman et al., 1992). The area under the ROC curve (AUC) is a widely used metric in the evaluation of species distribution models (Townsend Peterson et al., 2007; Jiménez-Valverde, 2012). The AUC is generally in the range of 0.5 to 1. The larger the value, the more accurate the prediction. Usually, a value between 0.9 and 1 is very good (Swets, 1988). For future climate projections, we used two representative concentration pathways (RCPs) of the IPCC-RCP 2.6 and 8.5 (GCM: BCC-CSM2-MR) for two future periods, 2050 and 2070. To explore the direction of the core distributional shifts between current and future ranges, we summarized the distribution to a single point, the centroid of suitable distribution (Vanderwal et al., 2013; Brown, 2014). We determined the migration direction of the geographical centroids for *S. alterniflora* under different RCP conditions and different periods by ArcGIS 10.4.1 (Yu et al., 2019; Tang et al., 2021b).

## Results

### The Historical Expansion Process of *S. alterniflora*

**Figure 1:**
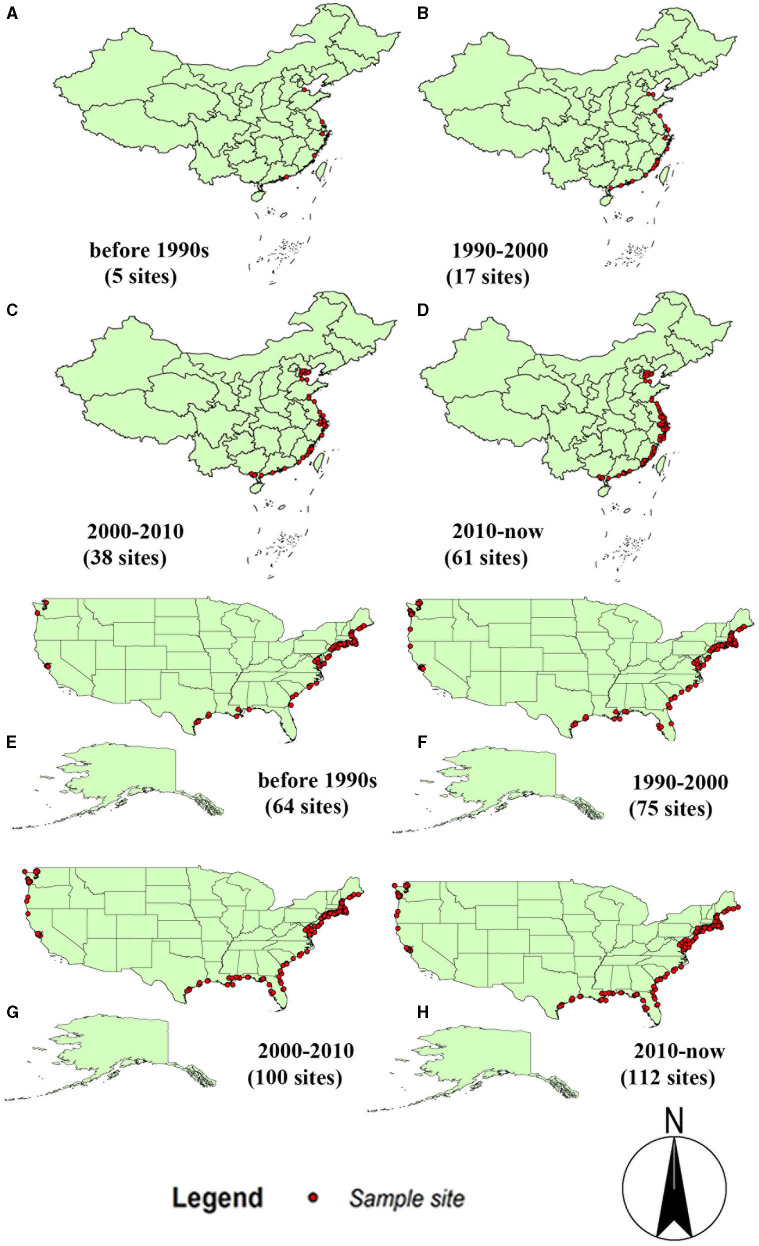
The dynamic map of *Spartina alterniflora* in China and America. **(A)** Before the 1990s in China; **(B)** 1990–2000 in China; **(C)** 2000–2010 in China; **(D)** 2010–now in China; **(E)** before the 1990s in America; **(F)** 1990–2000 in America; **(G)** 2000–2010 in America; **(H)** 2010–now in America.

*Spartina alterniflora* was produced on the southeastern coast of America. It was introduced to China as early as 1979 and then spread to the whole country. After more than 30 years of development, *S. alterniflora* has spread throughout the coastal areas of China. It can be seen from Figures 1A–D that *S. alterniflora* extends from the central coast to both sides of the coast in China, while it extends from the west coast to the east coast in America (Figures 1E–H). From the expansion trend of the two countries, *S. alterniflora* grows along the coastline and is the dominant plant in the coastal intertidal zone.

When invasive alien species enter a new area and period after establishing the population, there is often little or no spread. This stage is called the lag period. After the lag period, the distribution area of invasive species will spread on a large scale. Only when a natural barrier (such as mountains, oceans, etc.) appears in the space where the population spreads, the spread will slow down and gradually approach a specific level, which has reached the saturation period (Shigesada and Kawasaki, 1997). The *S. alterniflora* spread in China satisfies this viewpoint. Prior to the year 2000, *S. alterniflora* had been in a lag period, and after 2000, it showed a large-scale spread. In Figures 1B–D, it can be seen that *S. alterniflora* occurred in 17 sites in China from 1990 to 2000, and its occurrence increased to 38 sites from 2000 to 2010, and up to 61 sites from 2010 to now.

### Direct Comparison of Environmental Variables Between Origin and Invasion Countries

**Figure 2:**
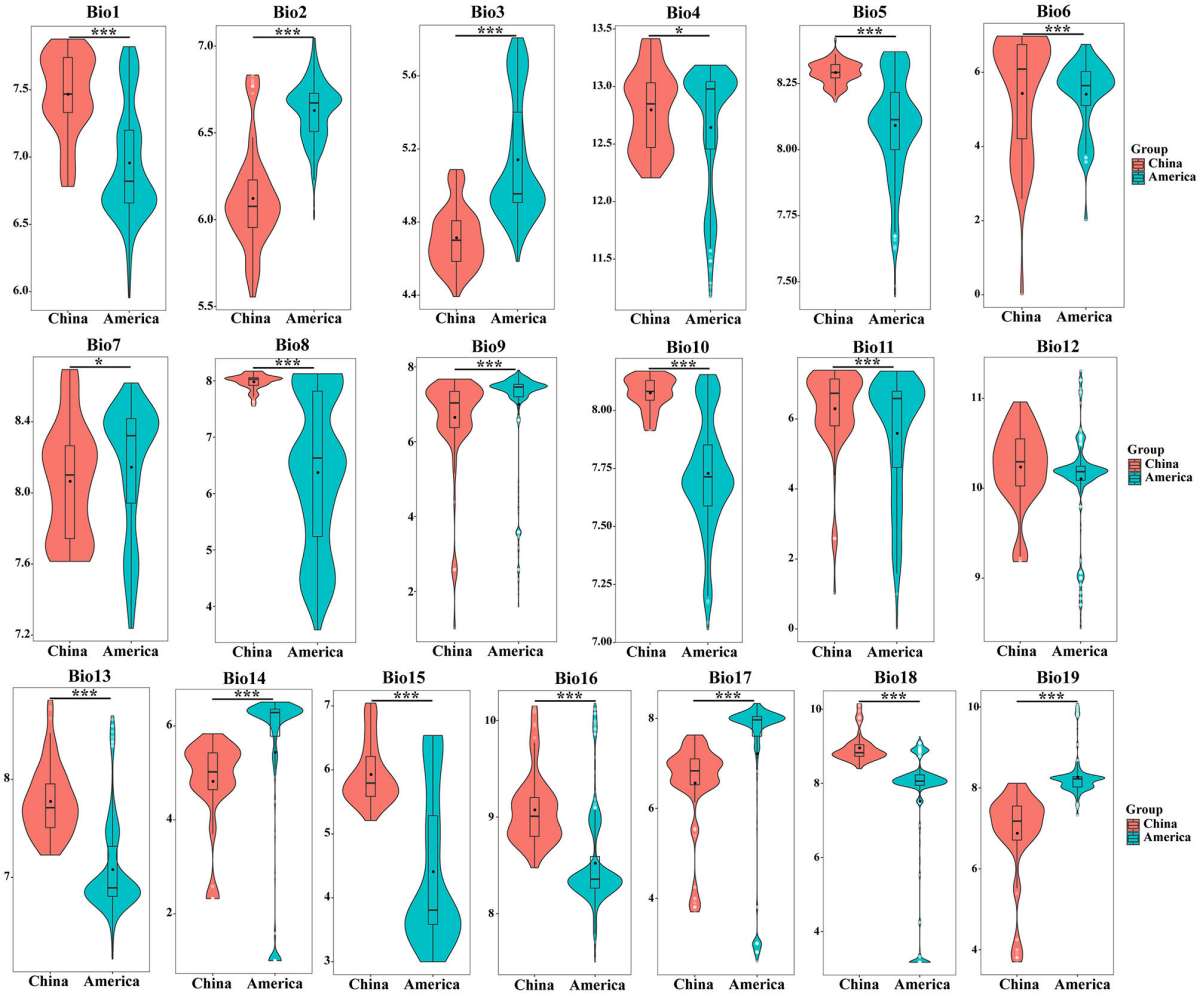
The comparison of *S. alterniflora* environmental variables between origin and invasion countries. The asterisk shows the level of significance between environmental variables in China and America. * means *P* < 0.05, ** means *P* < 0.01, *** means *P* < 0.001.

As shown in Figure 2, the average values of the 19 environmental variables of *S. alterniflora* in the origin and invasion areas are significantly different. Compared with the climatic variables of the origin and invasion areas, all climate variables except bio4, bio7, and bio12 have changed significantly (*P* < 0.001). The annual average temperature (bio1) of *S. alterniflora* in China is 5.083°C, which is higher than that of the country of origin, and the adaptable temperature of *S. alterniflora* in the invasion country is higher than that in the origin during the coldest year. Compared with the origin country, the minimum temperature of the coldest month (bio6) and mean temperature of the coldest quarter (bio11) increased by 5.555 and 4.393°C in the invasion country, respectively. At the hottest time of the year, *S. alterniflora* can adapt to higher temperatures than the place of origin, wherein the maximum temperature of the warmest month (bio5), the mean temperature of the wettest quarter (bio8), and the mean temperature of the warmest quarter (bio10) of the invasive population were 3.853, 3.561, and 5.497°C higher than those in the origin country, respectively. When the precipitation is the lowest in the year, the *S. alterniflora* invasion country can adapt to the drier environment compared with the origin country, wherein the humidity of the invasion country is 1.92–2.32 times lower than the origin in the precipitation of the driest month (bio14), the precipitation of the driest quarter (bio17), and the precipitation of the coldest quarter (bio19). Overall, *S. alterniflora* is more and more adapted to the high temperature and dry environment in the invasion country.

### Climatic Niche Overlap, Equivalency, and Similarity

**Figure 3:**
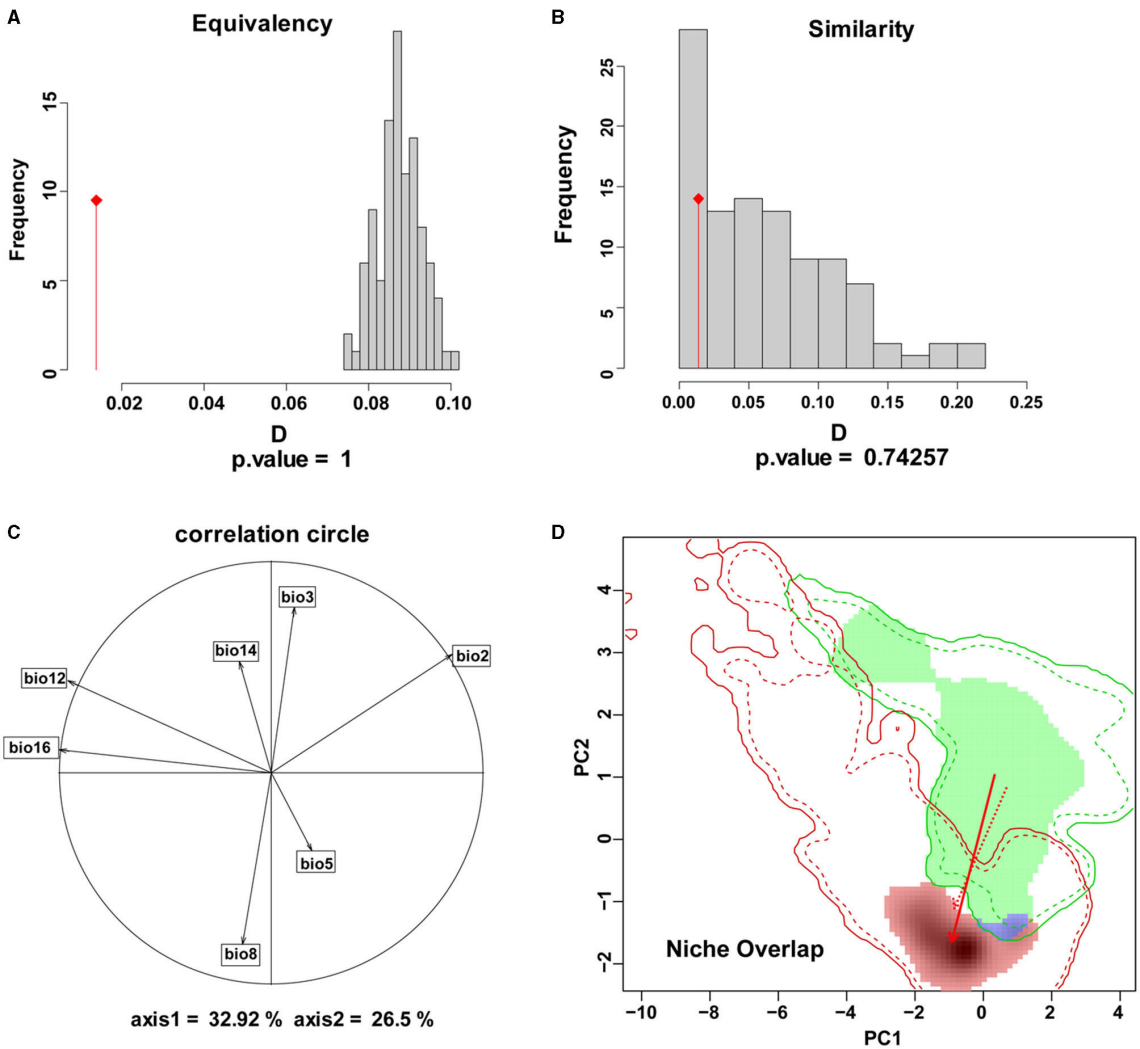
The niches of *S. alterniflora* in China and America under climatic ecological space. **(A)** Represents the contribution of each variable to the principal component axis, **(B, C)** represent the histograms representing the niche equivalence **(D)** test and niche similarity test of the two regions. **(D)** Indicates the overlapping of the species origin and invasion countries. Blue indicates niche overlap, green represents unfilling, and red represents expansion.

**Figure 4:**
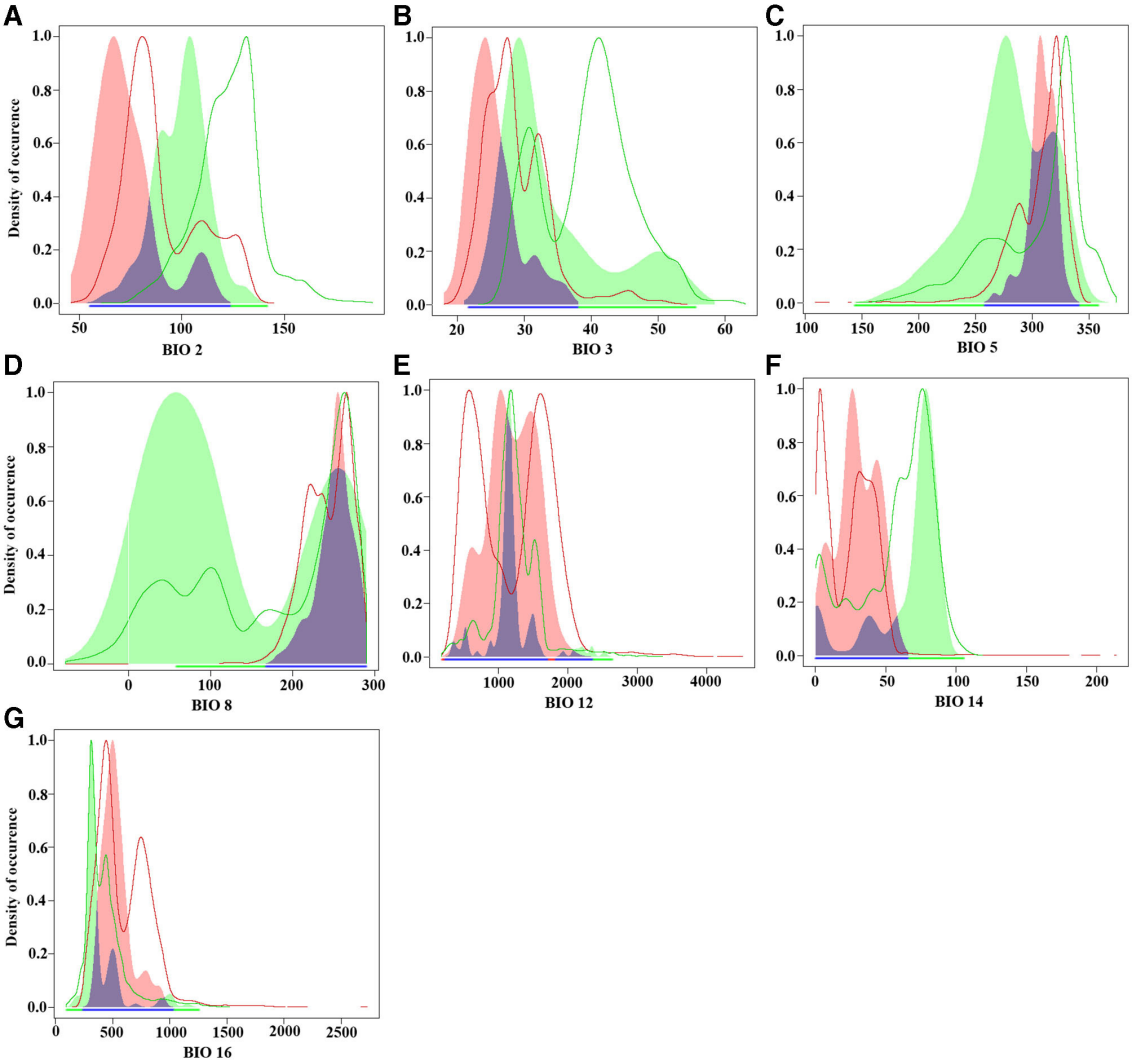
Predicted niche occupancy (PNO) profiles. Blue indicates niche overlap, green represents the origin country, and red represents the invasion country. **(A)** Mean diurnal range (Bio2); **(B)** isothermality (Bio3); **(C)** max temperature of the warmest month (Bio5); **(D)** mean temperature of the wettest quarter (Bio8); **(E)** annual precipitation (Bio12); **(F)** precipitation of the driest month (Bio14); **(G)** precipitation of the wettest quarter (Bio16).

The results of the comparative analysis of the climatic niche of *S. alterniflora* in the invasion country, China, and the origin country. America, are shown in Figures 3, 4. The PCA results of the climate variables selected by the MaxEnt model showed that the first two principal components can explain 59.42% of each parameter variable (PC1 = 32.92%, PC2 = 26.5%), and the realized climatic niche of *S. alterniflora* may have shifted and expanded in the invasive regions examined. The first axis (32.92%) was best explained by a negative correlation with climate variations, while a positive correlation best explained the second axis (26.5%) with climate variations. The direction of the center shift between the climate niche of the origin country, America, and the invasion country, China, is different from the background climate change of its two regions, indicating that the actual distribution of *S. alterniflora* in China and America occupies a more significant proportion than that of the origin country, as the center of the realized climatic niche moved toward warmer temperatures and lower humidity. There was only a 0.4% niche overlap between native and invaded ranges. The PNO profiles indicated differences in the climatic requirements of *S. alterniflora* between native and non-native countries (Figure 4). The potential distribution of *S. alterniflora* in the invaded range showed more excellent suitability in drier areas (Figures 4E,F), with a small mean diurnal range (Figure 4B). On the contrary, the potential distribution in the native range noted more excellent suitability in wetter environments.

The niche overlap indicates the similarity of the use of different environmental resources by the same species: the size of the niche overlap reflects the similarity of plant utilization resources; a large niche overlap indicates that their ecological requirements are close, the utilization of resources is similar, and the biological characteristics are similar under certain environmental conditions. However, the niche overlap between China and America is only 0.4%, which indicates that there is a significant difference between the native and invaded niches of *S. alterniflora*. The biological characteristics of *S. alterniflora* are not similar in the two countries, the requirements for the ecological environment are also not close, and the niche shift is significant.

### Potential Ecologically Suitable Distribution of *S. alterniflora* in Invasion and Origin Countries Under Current Climate Conditions

**Figure 5:**
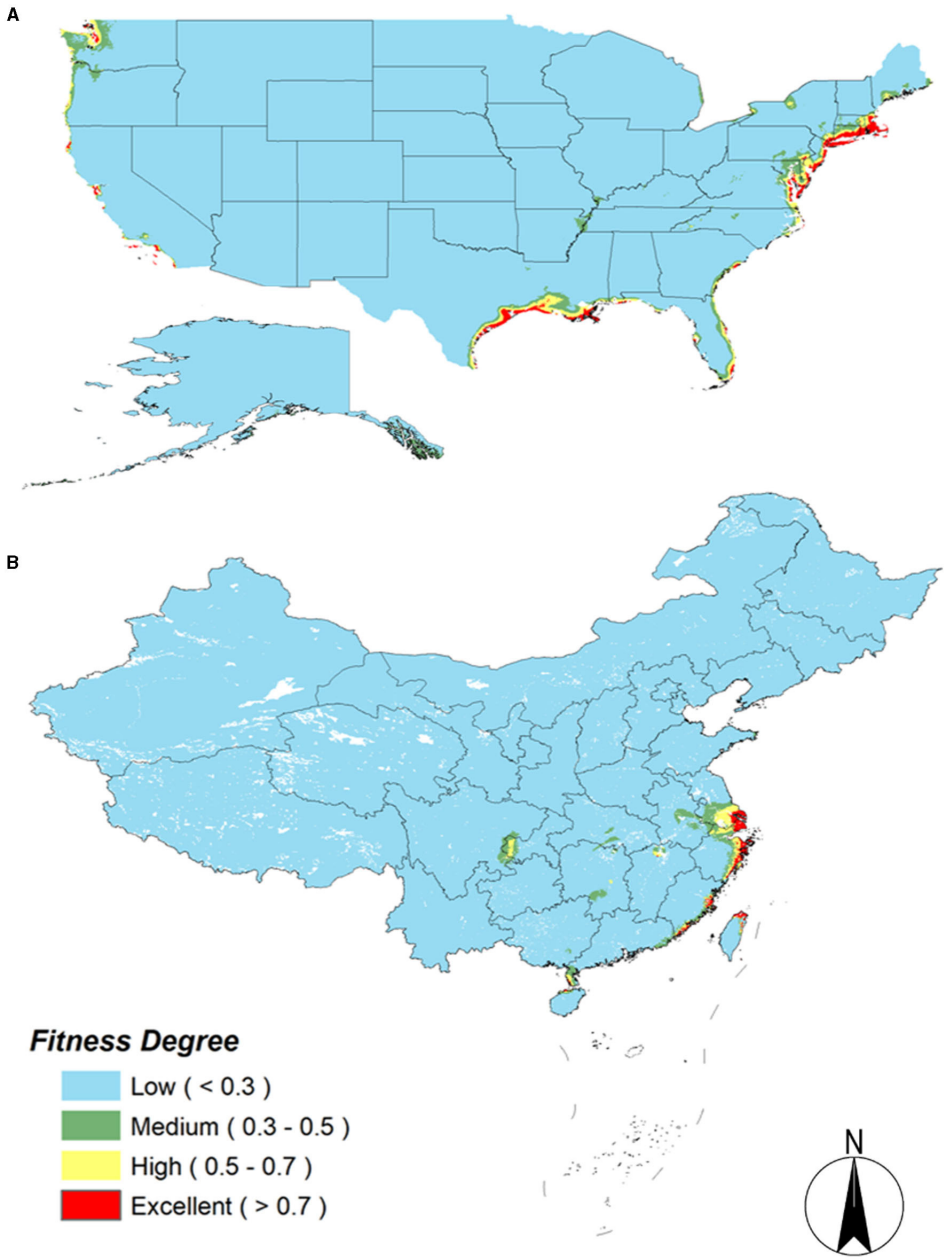
Potential distribution of *S. alterniflora* in China and America. **(A)** America; **(B)** China.

The area under the ROC curve of the MaxEnt model has an AUC value of 0.981, indicating that the constructed model has a good predictive ability and can reasonably predict the potential habitat of *S. alterniflora* in China and America. The predicted value of the model is divided according to the operation data to generate the ecologically suitable level distribution map. Red indicates the area that is the most suitable for *S. alterniflora* to grow. As shown in Figure 5A, the potential ecological suitable distribution of *S. alterniflora* in the United States is mainly distributed on the east and west coasts. The northeast coast along the Gulf of St. Lawrence and the southern Mexico Gulf and a small amount of the California coast exist as the most suitable distribution areas. Suitable distribution areas also exist along the coasts of Washington and Oregon. As shown in Figure 5B, the prediction results of *S. alterniflora* in the ecological suitable distribution of China show that, under current climatic conditions, the Jiangsu, Shanghai, Zhejiang, and Fujian coastal areas and the northern Taiwan provinces all have ecological distribution areas most suitable for *S. alterniflora*.

### Potential Ecologically Suitable Distribution of *S. alterniflora* in Invasion and Origin Countries Under Future Climate Conditions

**Figure 6:**
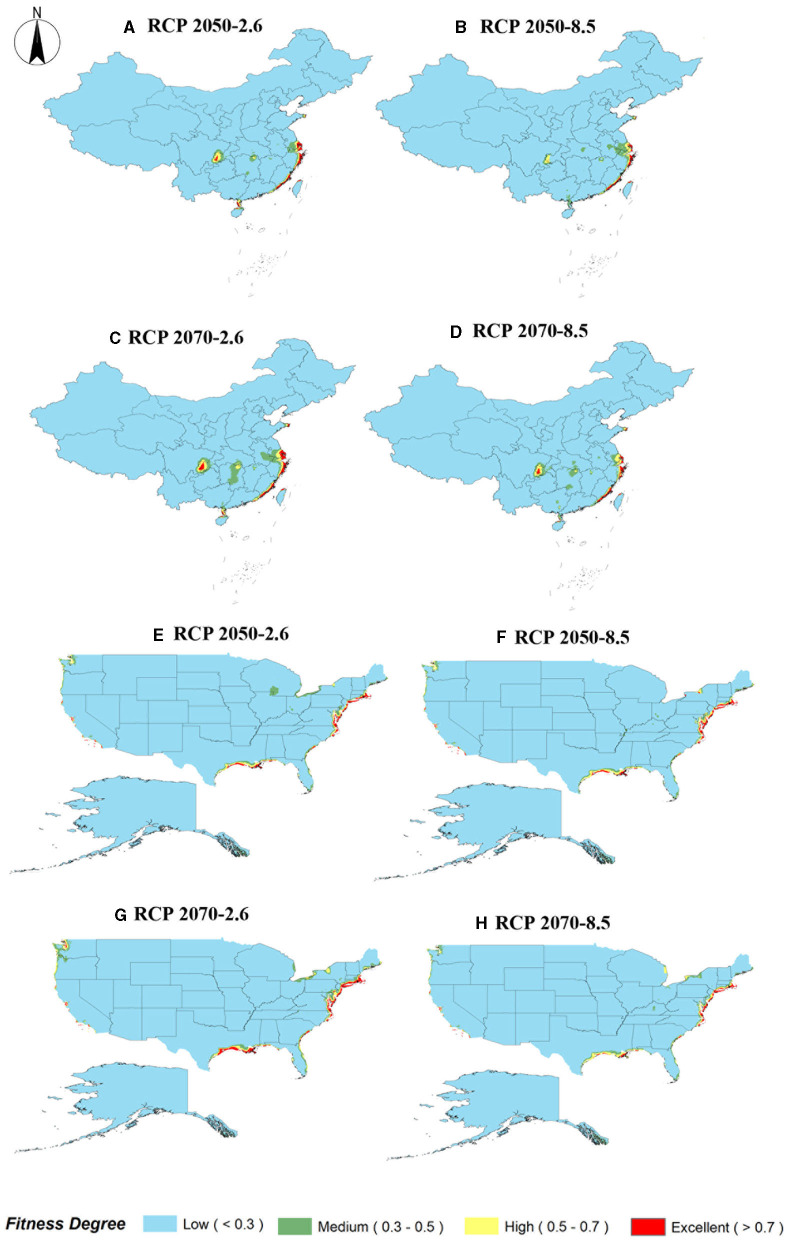
Future species distribution models of *S. alterniflora* in China and America under different climate scenarios predicted by MaxEnt. **(A–D)** RCP 2050-2.6 in China; RCP 2050-8.5 in China; RCP 2070-2.6 in China; RCP 2070-8.5 in China; **(E–H)** RCP 2050-2.6 in America; RCP 2050-8.5 in America; RCP 2070-2.6 in America; RCP 2070-8.5 in America.

**Figure 7:**
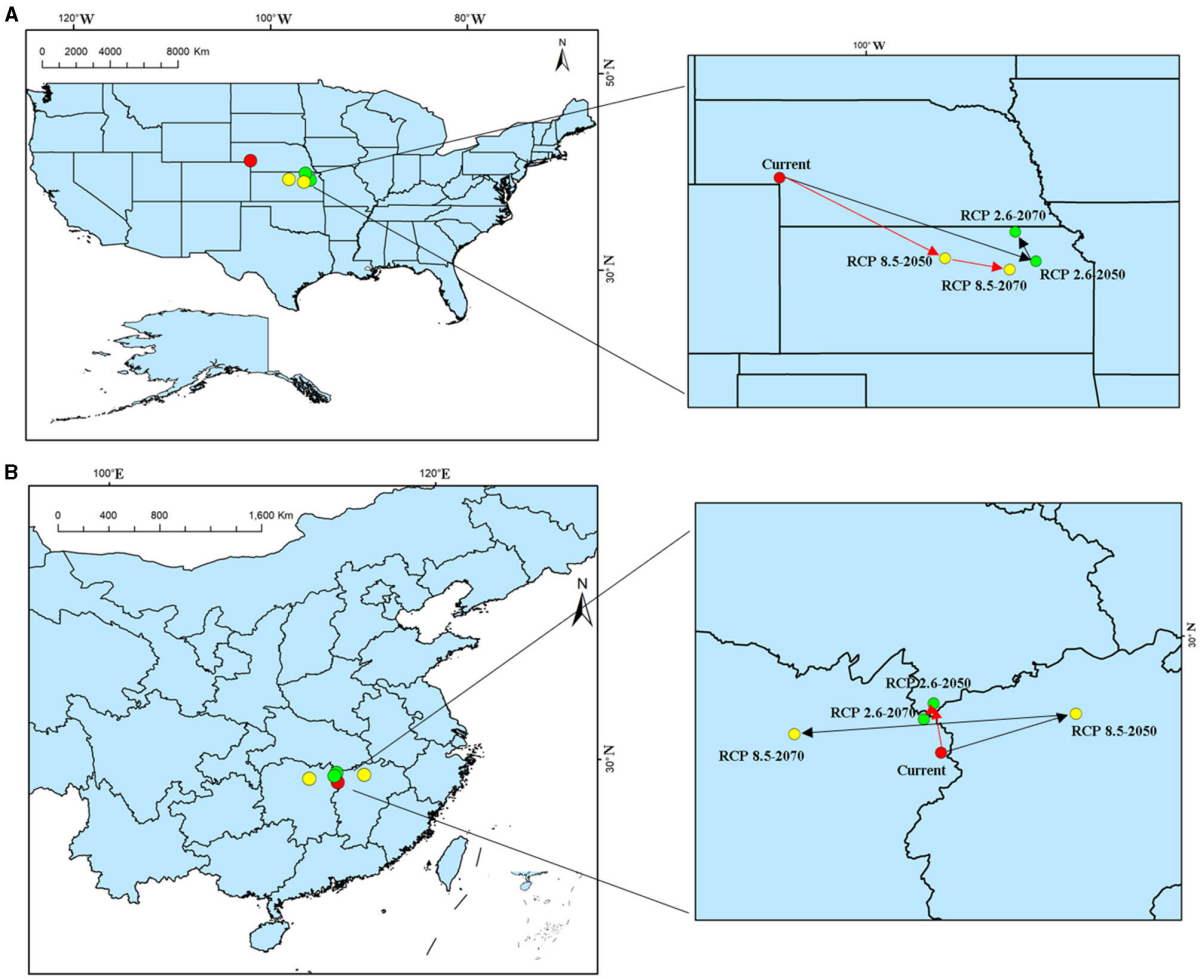
Geometric central migration of *S. alterniflora* habitat suitability areas in 2050 and 2070 under two distinct climate change scenarios. **(A)** America; **(B)** China.

The analysis and prediction of the potential suitable distribution of *S. alterniflora* under the two greenhouse gas emission scenarios (RCP 2.6 and RCP 8.5) in the 2050s and 2070s are shown in Figure 6. In China and America, under the greenhouse gas emission scenarios of RCP 2.6 and RCP 8.5, the ecologically suitable distribution areas for *S. alterniflora* will be mainly concentrated in coastal areas. By 2070, the suitable area of *S. alterniflora* in the inland areas of China will increase and be concentrated in the Sichuan–Chongqing junction and Hunan provinces. Under RCP 2.6, the ecologically suitable distribution areas of *S. alterniflora* in China are growing slowly until 2050, and will be rapidly growing to 2070. Under RCP 8.5, the ecological suitable distribution areas of *S. alterniflora* in China are expected to grow slowly from now to 2070. Furthermore, *S. alterniflora* ecological suitable distribution areas are relatively rare in America, only existing in the junction of Wisconsin and Iowa under RCP 2.6 in 2050. From the changes in the area of the invasion and origin countries, even if the area of the invasion country increases, it will always be smaller than the area of the country of origin. The trends of the area increase and decrease of the invasion country, China, and the origin country, America, are consistent (Table 2). The change of centroids reflects the directional shift of future ranges (Figure 7). Under different climate change scenarios, the direction changes of *S. alterniflora* are simulated to be quite different. In America, its range is expected to shift to the southeast under RCP 2.6 and RCP 8.5. In China, its range is expected to change to the north under RCP 2.6, but under RCP 8.5, the centroids shift to the east and then shift to the west.

**Table 2:** The total area in China and America with *S. alterniflora* ecological suitable distribution levels > 0.3 under RCP 2.6 and 8.5.

**Country**	**Level**	**0.3–0.5**	**0.5–0.7**	**0.7–1**	**Total**	**Change**
China	Current	9.214 ×10^4^ km^2^	3.753 ×10^4^ km^2^	2.511 ×10^4^ km^2^	15.478 ×10^4^ km^2^	**–**
	RCP2.6-2050	9.766 ×10^4^ km^2^	4.655 ×10^4^ km^2^	3.561 ×10^4^ km^2^	17.982 ×10^4^ km^2^	16.2%
	RCP8.5-2050	10.490 ×10^4^ km^2^	3.845 ×10^4^ km^2^	2.262 ×10^4^ km^2^	16.597 ×10^4^ km^2^	7.2%
	RCP2.6-2070	19.163 ×10^4^ km^2^	6.816 ×10^4^ km^2^	4.832 ×10^4^ km^2^	30.811 ×10^4^ km^2^	99.1%
	RCP8.5-2070	11.653 ×10^4^ km^2^	5.300 ×10^4^ km^2^	2.958 ×10^4^ km^2^	19.911 ×10^4^ km^2^	28.6%
America	Current	22.771 ×10^4^ km^2^	9.488 ×10^4^ km^2^	6.823 ×10^4^ km^2^	39.082 ×10^4^ km^2^	**–**
	RCP2.6-2050	15.511 ×10^4^ km^2^	6.385 ×10^4^ km^2^	5.107 ×10^4^ km^2^	27.003 ×10^4^ km^2^	−30.9%
	RCP8.5-2050	12.212 ×10^4^ km^2^	7.462 ×10^4^ km^2^	5.104 ×10^4^ km^2^	24.778 ×10^4^ km^2^	−36.6%
	RCP2.6-2070	16.335 ×10^4^ km^2^	8.741 ×10^4^ km^2^	8.167 ×10^4^ km^2^	33.243 ×10^4^ km^2^	−14.9%
	RCP8.5-2070	13.767 ×10^4^ km^2^	7.821 ×10^4^ km^2^	3.723 ×10^4^ km^2^	25.311 ×10^4^ km^2^	−35.2%

## Discussion

*Spartina alterniflora* was introduced to China in 1979 and then spread rapidly along the coastal beach. So far, the distribution of *S. alterniflora* spans 19 latitudes, from Liaoning in the north to Guangdong and Guangxi in the south (An et al., 2007; Zuo et al., 2012). China has become the country with the largest outbreak of *S. alterniflora*. The extensive spread of *S. alterniflora* has threatened the coastal wetland ecosystem of China. According to the *S. alterniflora* invasion and diffusion dynamics, the *S. alterniflora* spread in China can be divided into two stages: The first stage was from 1979 to 2000, which was the sporadic stage of *S. alterniflora*. *Spartina alterniflora* was concentrated in the coastal areas of Jiangsu, Zhejiang, and Fujian with earlier invasion times. At this stage, *S. alterniflora* invaded, successfully colonized, and spread to the surrounding areas of the invaded area. The second stage is from 2000 to the present. It is the rapid diffusion stage of *S. alterniflora*, showing a gradual spread from the coastal areas to the coastal provinces, and the distribution range gradually expanded.

The *S. alterniflora* invasion and spread pattern in China is from the central coast to the north–south coast. It may be because *S. alterniflora* was first introduced in the Fujian province, and gradually spread from the middle to the north and south. According to the literature, *S. alterniflora* has a robust sexual reproduction ability in North America, which is related to its geographical distribution and plays an important role in population spread and invasion (Callaway and Josselyn, 1992; Plyler and Carrick, 1993). In addition, *S. alterniflora* seeds have extremely strong salt tolerance characteristics and maintain high activity after soaking in 2–4°C seawater for 3 months, and their germination ability reaches the maximum (Mooring et al., 1971). This is an excellent adaptation to the salinity environment of the coastal wetland, which increases the opportunities for competition and spread and the invasion and spread of populations (Wijte and Gallagher, 1996). Studies in Willapa and San Francisco Bay in the United States have confirmed that the establishment and spread of *S. alterniflora* populations in this area are mainly through the propagation and spread of seeds. The propagation of germs is the most essential way for *S. alterniflora* populations to spread (Daehler, 1999; Davis et al., 2004).

Using *S. alterniflora* environmental variable data in China to compare with the origin country in America, it is found that the niche overlap between the climate niche of the origin country and the niche of the invasion country is small, indicating a relative lack of analog climates. Therefore, the species in the invaded area occupy non-native niches because the background in China is different than in America. The stability value reflects the conservation of the ecological niche of the species, while the expansion value reflects the new environment occupied by species (Broennimann et al., 2014; Callen and Miller, 2015). During the invasion of China, its niche needed to be changed significantly in the climate space. It shows that the niche demand of *S. alterniflora* in the invasion country has changed dramatically. As it is introduced into China, its niche demand is significantly different from the origin in the climate space. During the invasion process, several factors will lead to a niche shift. In turn, these factors will promote the colonization of new combinations of environmental conditions, causing species to exceed their native niche limits and occupy different non-native niches (Nunes et al., 2015). These factors include abiotic differences, biological interactions, decentralized barriers, or human activities (Wan et al., 2017). Climate differences can play an important role in ecological differences by promoting species to adapt to the new climate environment (Graham et al., 2004; Rissler and Apodaca, 2007).

The geographical and climatic conditions of the invaded areas and their spread patterns can often be used as an essential reference for predicting the suitable habitat of alien invasive plants (Kriticos and Randall, 2001). A species can cause the same damage in two places with very similar ecological conditions, so the case for the species itself is the best predictor. To predict the potential distribution of an alien species, it is necessary to analyze their current distribution and the climatic and environmental conditions of the invasion area (Kriticos and Randall, 2001). Therefore, this study attempted to predict the potential distribution of *S. alterniflora* in China and America by using the niche model software based on the invasion and diffusion patterns of *S. alterniflora* during historical reconstruction.

Based on the data obtained from specimen records and literature reports, we obtained the potential distribution area of *S. alterniflora* in China using the MaxEnt model. It can be seen that *S. alterniflora* is suitable for growing in coastal areas. The range of suitable habitats currently distributed in China is smaller than that predicted by MaxEnt. Therefore, *S. alterniflora* has room and possibility to continue to spread in China. However, the predictions in the United States were different. Regardless of whether they were under RCP 2.6 or RCP 8.5, *S. alterniflora* was predicted to have a smaller suitable area in the United States than currently; therefore, it is less likely that *S. alterniflora* will continue to expand in the United States. Climate is an essential factor affecting the distribution of *S. alterniflora* on a large scale. *Spartina alterniflora* exhibits different adaptive characteristics under different latitudes and climatic conditions, and its distribution range also changes (Zhao et al., 2015). This showed that *S. alterniflora* has strong climate adaptability. Therefore, *S. alterniflora* could still have a significant expansion trend in China in the case of small niche overlap between China and the United States.

## Conclusions

Our study provided strong evidence of a shift in the realized climatic niche of *S. alterniflora* during its spread through China. This study analyzed the primary process and the earliest invasion area of *S. alterniflora* in China by reconstructing the historical process of its spread. Furthermore, we compared the climate adaptability of the *S. alterniflora* origin and invasion countries and analyzed the climate adaptability characteristics of *S. alterniflora* after it invaded China. Finally, based on the climate adaptability of *S. alterniflora* in China and its climatic adaptability during the invasion, the MaxEnt model and ArcGIS were used to predict its suitable ecological distribution in China and America, and monitoring and early warning were conducted to control the further spread of *S. alterniflora* in China. The results showed that *S. alterniflora* spreads from the middle coast to both sides of the coast in China, while it spreads from the west coast to the east coast in America. By comparing 19 environmental variables of *S. alterniflora* in the invasion and origin countries, it was found that *S. alterniflora* is more and more adaptable to the high temperature and dry environment in the invasion country. Moreover, a comparison between the niches of China and America found that the niche overlap is small, indicating that the requirements for the ecological environment of *S. alterniflora* are not close, and the niche shift is large.

## Data Availability Statement

The original contributions presented in the study are included in the article/supplementary material, further inquiries can be directed to the corresponding author.

## Author Contributions

YY and XT: conception and design of the research. XT: acquisition of data. YY: analysis and interpretation of data. ML and XL: statistical analysis. YY and JT: drafting the manuscript. All authors contributed to the article and approved the submitted version.

## Funding

This project was supported by the National Natural Science Foundation of China (Grant No. 41901375), Hebei Natural Science (Grant No. D2019209322), and Program for the Youth talent of Higher Learning Institutions of Hebei National Special Fund for Forestry Scientific (Grant No. BJ2020058).

## Conflict of Interest

The authors declare that the research was conducted in the absence of any commercial or financial relationships that could be construed as a potential conflict of interest.

## Publisher's Note

All claims expressed in this article are solely those of the authors and do not necessarily represent those of their affiliated organizations, or those of the publisher, the editors and the reviewers. Any product that may be evaluated in this article, or claim that may be made by its manufacturer, is not guaranteed or endorsed by the publisher.
